# Analyzing the structural, optoelectronic, and thermoelectric properties of InGeX_3_ (X = Br) perovskites via DFT computations

**DOI:** 10.1038/s41598-024-72745-w

**Published:** 2024-10-09

**Authors:** Danish Abdullah, Dinesh C. Gupta

**Affiliations:** Condensed Matter Theory Group, School of Studies in Physics, Jiwaji University, Gwalior, 474011 India

**Keywords:** Non-magnetic semiconductors, Mechanical stability, Direct bandgap, Optoelectronic device applications, Thermoelectric features, Materials science, Physics

## Abstract

The Electronic and optical properties of InGeX_3_(X = Cl, Br) were examined by adopting the density functional theory (DFT) approach. We applied the GGA + Trans-Blaha modified Becke–Johnson (TB-mBJ) technique to acquire the precise bandgap of 1.52 and 0.98 eV of the compounds InGeX_3_(X = Cl, Br) respectively which suggests the direct bandgap at (M-M). The stability of the material is confirmed by the formation energy (– 2.83 = Cl; – 2.35 = Br) and Mechanical stability. Primarily elastic constants were extracted for each of the materials under scrutiny, and these values then served to gauge all of the materials’ mechanical properties. The assessed Poisson’s and Pugh’s ratios for the materials InGeCl_3_ and InGeBr_3_ were verified to identify the degree of ductility. The quasi-harmonic Debye model additionally covers the temperature and pressure dependence on thermodynamic parameters, particularly volume, specific heat capacity (Cv) at constant volume, and the Gruneisen parameter (γ) in the range of 0–800 K and 0–5 GPa. It is anticipated that InGeCl_3_ and InGeBr_3_ will have static dielectric constants of 4.01 and 5.74, respectively. InGeX3(X = Cl, Br) also reveals significant absorption in the high UV spectrum. The thermoelectric properties have also been calculated vdata-element-id="9QNfR3VHbcMHX_W0fJCYp" data-element-type="html" style="display: initial; visibility: initial; opacity: initial; clip-path: initial; position: relative; float: left; top: 0px; left: 0px; z-index: 1 !important; pointer-events: none;" />ia boltztrap2 code using a k mesh of around 1,50,000 points.

**Supplementary Information:**

The online version contains supplementary material available at 10.1038/s41598-024-72745-w.

## Introduction

The formula ABX_3_ materials have grabbed an abundance of discussion due to their configurable optical bandgap^1,2^, long electron-hole diffusion length generation potential^3,4^, and affordable price^5^. Consequently, these materials display significant charge carrier mobility and are very cognizant of electromagnetic radiation. Perovskite crystals are often used as the material for light-emitting diodes (LEDs) and sensors^6–9^. Additionally, they are credible contenders for the future generation of solar cells that exist commercially. Due to its high efficiency and inexpensive cost of manufacturing, perovskite solar cells (PSCs) have become one of the most intriguing alternatives to conventional silicon-based solar cells in recent years. Safe inorganic metal halide perovskite materials have become of vital significance for a wide range of applications, from photovoltaics to optoelectronics and even more. Their extraordinary optoelectronic characteristics affordability and environmental safety make them indispensable in the hunt for environmentally conscious reliable technology. Around the world, Pb-based halide PSCs are already the most efficient kind of PSCs. Unfortunately, concern about the renowned toxicity of Pb in these devices has hindered their widespread manufacturing and commercialization^10–19^. As a result, finding lead-free PSCs that are safe for the environment is a prerequisite to their widespread production. Several innovative lead-free perovskite materials have been assessed computationally and experimentally to alleviate this demand. Minimal toxicity, small and direct bandgaps, strong optical absorption coefficients, high mobilities, prolonged charge-carrier lifetimes, modest exciton-binding energies, and excellent stability are some of the fundamental attributes of ideal Pb-free materials that work as absorbents in PSCs^20–23^. Recently, several potentially Pb-free materials have been encountered as ecologically friendly PSCs. These materials comprise certain double perovskites^24–27^, Sn-based perovskites^28–30^, Ge-based perovskites^31–33^, Bi-based compounds^32,34^, and Sb-based compounds^35,36^. Researchers have emphasized Sn- and Ge-based perovskites among these alternatives owing to Pb-based compounds and their comparatively higher efficiency than other Pb-free compounds.

According to the literature review, TlGeI_3_ and TlSnI_3_ have higher static dielectric constants, which argues that by boosting their absorption and efficiency, both of these materials will improve the operation of optoelectronic devices^37^. Additionally, it has been found that the cubic TlBX_3_ (B = Ge, Sn; X = Cl, Br, I) perovskite materials portray excellent optical properties, which render them ideal for use in solar cells, light-emitting diodes (LEDs), photodetectors, and other devices^38^. TlGeCl_3_ and TlSnCl_3_ possess significant absorption in the high ultraviolet range. Moreover, InSnX_3_ (X = Cl, Br, I)^39^ has been examined via a DFT-based first-principles calculation. The findings suggested that the materials are naturally ductile as well as stable in terms of chemically, mechanically, and thermodynamically. Interestingly, the perovskite materials have small bandgaps, and when Br and I were substituted for Cl, a shift from an indirect to a direct bandgap was seen. Additionally, the perovskites have remarkable optical qualities that render them perfect for use in solar cell technology. According to Soukaina Bouhmaidi et al., TlGeX_3_ (X = Cl, Br, and I)^40^ perovskites are direct semiconductors with superior optical characteristics, such as low reflection, high conductivity, and high absorption. These indicate the optoelectronic applications of the materials are very exciting and many other similar lead-free semiconductors^41–43^. Additionally, Sajid Khan et al. adopted density functional theory (DFT) to explore the structural, electronic, and optical aspects of inorganic indium-based halide perovskites with composition InACl_3_ (A = Ge, Sn, and Pb)^44^. The optimized lattice constants of these cubic compounds have been determined to be between 5.53 and 5.56 Å. InGeCl_3_ possesses a direct bandgap, but InSnCl_3_ and InPbCl_3_ have an indirect bandgap, based on the predicted band structure. For InGeCl_3_, InSnCl_3_, and InPbCl_3_, the energy band gap values are 1.89 eV, 0.87 eV, and 1.84 eV, respectively. Because of this, InGeCl_3_ is a desirable substitute for Pb in photovoltaic applications. The appropriateness of InSnCl_3_ and InPbCl_3_ for solar cells is demonstrated by their semiconducting energy gaps. Furthermore, utilizing the WIEN2k code inside density functional theory DFT, Abrar Nazir et al. disclosed the structural, electrical, lattice dynamics, optical, elastic, and thermoelectric characteristics of Indium-based Perovskite InGeX_3_(X = F, Cl)^45^. Pugh’s ratio characterizes the compounds’ ductile nature, while the Cauchy pressure test determines their ionic nature. These compounds may make suitable candidates for optoelectronic devices due to their broad-range absorption in the UV spectrum.

Inspired by the above-mentioned perovskites we carefully examined the structural, mechanical, and optoelectronic properties of single halide perovskite InGeX_3_(X = Cl, Br) which paves the way to photovoltaic and optical applications.

## Computational methodology

Computation techniques based on density functional formalism are approached to investigate these halide perovskites InGeX_3_(X = Cl, Br). The Wien2k-simulation code^46^ with a linearized augmented plane-wave basis set and potential from the core as well as valance electrons are implemented to solve the Kohn-Sham (KS) equation^47^ and define ground state charge density. For the iterative solution of the KS equation, the exchange-correlation potential is approximated by the generalized gradient approximation^48^ parameterized by Perdew-Burke-Ernzerhof. However, GGA undervalues the bandgap we have facilitated GGA by modified exchange potential earlier defined by Becke-Johnson^49^. The convergence of charge and energy is done up to 0.0001 and 0.0001Ry, respectively. The unit cell volume is shared among muffin tin spheres with radii and space in between muffin spheres. The wave function with muffin spheres is expanded as an atomic-like wave function with limit lmax = 10, while the plane wave basis set for the interstitial space is limited by setting R_MT_Kmax = 7. The k-sampling in the first BZ for the better convergence of the energy was set to 3000 integration points.

### Structural properties

**Fig. 1:**
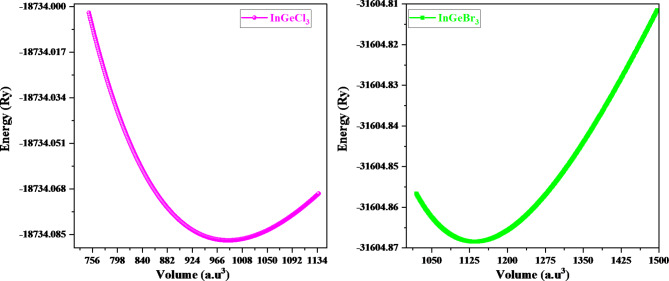
Optimization plot of investigated double perovskites InGeX_3_ (X = Cl, Br).

**Fig. 2:**
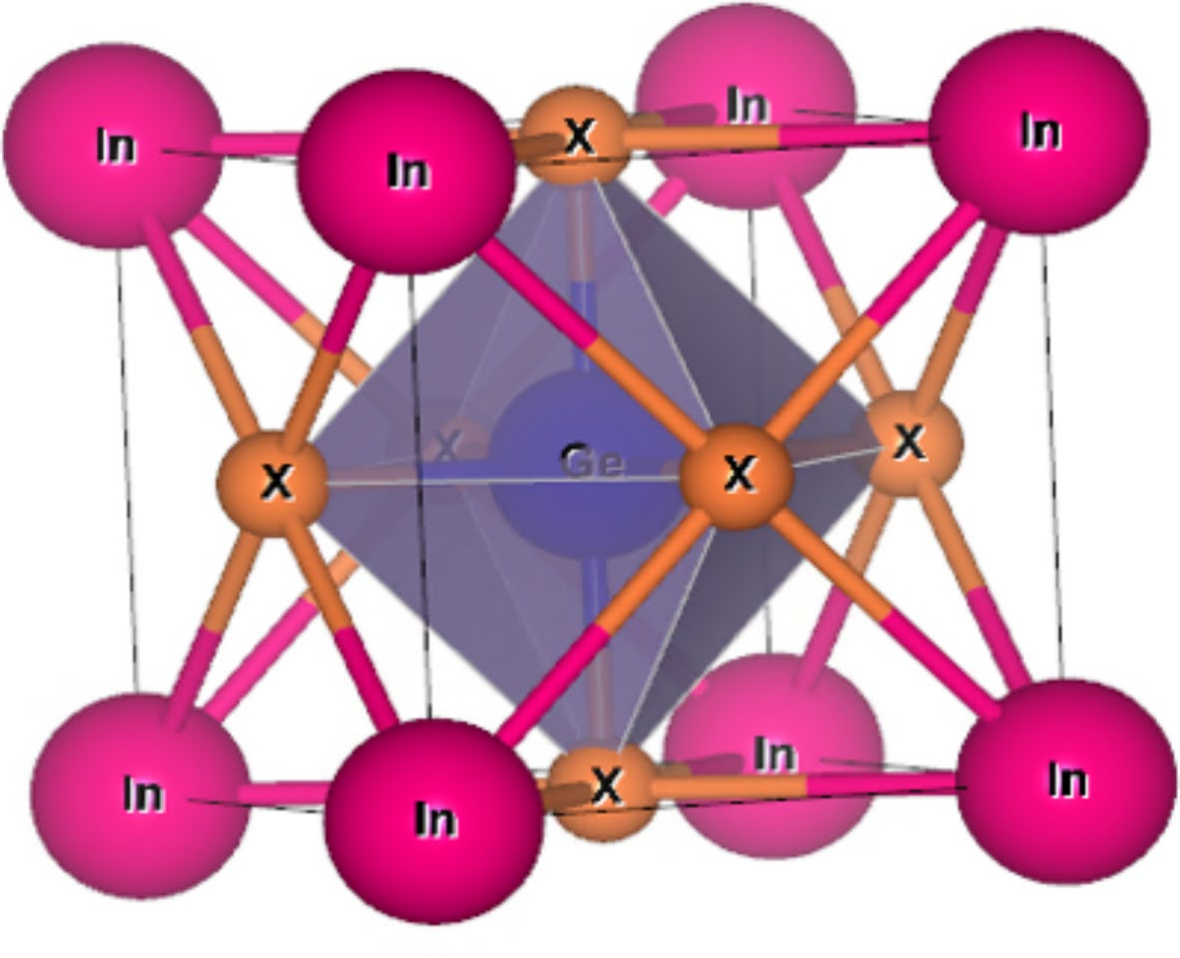
Cubic unit structural Cell of InGeX_3_ (X = Cl, Br).

The structure of InGeX_3_(X = Cl, Br) is predicted here employing GGA approximations, since, InACl_3_(A = Ge, Sn, Pb)^44^ is reported in cubic *pm-3 m* structure. Therefore, we have first computed the tolerance factor (τ)^50^ for these materials. The tolerance factor provides information about the most stable structural phase for these halide perovskites. The calculated values of the τ-factor of the given perovskites are given in Table 1, thereby suggesting these materials are most stable in a cubic structure. The stability in the *pm-3 m* cubic structure is further cross-verified by structural optimization of the titled material in the cubic structure. By employing simulation, the robustness and ground-state structure of any material can be assessed by reducing its energy to volume. The most likely state in the curve where the energy is lowest reflects the stability of the given structure. Figure 1 displays the minimum in the E-V curve, which reflects ground-state energy. Figure 2 shows the crystal structure of these materials. The results of the structural optimization Parameters like lattice constant (a_0_), bulk modulus (B), and pressure derivative of the bulk modulus (B`) are determined and are summarized in Table 1. The cubic crystal structure of the cubic InGeX_3_ (X = Cl and Br) perovskites is composed of “In” atoms at (0, 0, 0), Ge at (0.5, 0.5, 0.5), and “X = Cl, Br” adopting the Wyckoff positions of (0, 0.5, 0.5), (0.5, 0, 0.5), or (0.5, 0.5, 0). Considering the Birch-Murnaghan equation of state (EOS)^51^, the basic structural characteristics are deduced from the estimated curves of the primitive unit cell’s total energy versus total volume. These materials’ bulk modulus (B) and stable structural lattice attributes boost their effectiveness, while a material’s pressure derivative of “B´” can be advantageous for identifying its thermoplastic properties. Therefore, it is fair to assess both parameters. The examined values of “B,” which decrease from InGeCl_3_ to InGeBr_3_, are shown in Table 1. InGeCl_3_ has a greater bulk modulus value than InGeBr_3_, suggesting that it is tougher than InGeBr_3_. Equation 1 has been employed to figure out the formation energy, or ∆*Ef*, which is the energy that emerged during the perovskites’ formation from their constituent atoms. It can be deployed for examining perovskites’ chemical stability. The negative value of the formation energy of InGeCl_3_ and InGeBr_3_ suggested its thermodynamical stability. The formation energy interpretation manifests as

1 $$\:\varvec{\varDelta\:}\varvec{E}\varvec{f}=\frac{{\varvec{E}}_{\varvec{T}\varvec{o}\varvec{t}}\left(\varvec{I}\varvec{n}\varvec{G}\varvec{e}{\varvec{X}}_{3}\right)-\varvec{E}\left(\varvec{I}\varvec{n}\right)-\varvec{E}\left(\varvec{G}\varvec{e}\right)\_3\varvec{E}\left(\varvec{X}\right)}{5}$$

where E_Total_ (InGeX_3_) is the total energy of perovskites and the energies E_In_, E_Ge_, and E_X_ (X = Cl, Br), correspond to In, Ge, and X elements.

**Table 1:** The determined values of lattice constant (a), volume(V), bulk-modulus (B), pressure derivative of bulk-modulus (Bˊ), ground state energy E_0_, formation energy (ΔEf = eV/atom) and tolerance factor (τ_F_) of InGeX_3_ (X = Cl,Br).

Compounds	a(Å)	V (a.u^3^)	B(GPa)	B`	E_0_	ΔE_f_	τ_F_
InGeCl_3_	5.2632	983.8541	28.02	4.33	–18734.087143	–2.83	0.90
InGeBr_3_	5.1595	1134.9536	24.20	4.38	–31604.868406	–2.35	0.91

### Mechanical stability

When implementing the material in execution, the mechanical qualities serve as a valuable tool. Three separate elastic constants, C_11_, C_12_, and C_44_, are commonly used to quantify the robustness of cubic crystals. If C_11_, C_12_, and C_44_ fulfill the following three conditions^52,53^, then the mechanical properties of the material are stable: C_11_ + 2 C_12_ > 0, C_44_ > 0, and C_11_ − C_12_ > 0. Table 2 displays the values of C_11_, C_12_, and C_44_ corresponding to InGeX_3_ (X = Cl, Br). Both of the compounds’ elastic constant values fulfill the above criteria confirming the mechanical stability of InGeX_3_ (X = Cl, Br) in equilibrium. By applying the elastic constants, which can be computed using equation two given below, we can figure out the anisotropy factor A for these compounds. The association between A and 1 generates a compound’s anisotropic properties. When the anisotropy factor A is equivalent to one, the material responds isotropically; otherwise, it performs anisotropically, and as the anisotropy factor A shifts away from one, the compound’s anisotropy becomes more apparent. The anisotropy factors for InGeCl_3_ and InGeBr_3_, which are 0.39 and 1.00, respectively. It may be guessed that InGeCl_3_ is anisotropic. The anisotropy factor A of InGeBr_3_ is one, signifying that these materials are virtually isotropic.

**Table 2:** Calculated values of elastic constants C_11_, C_12_, C_44_ in (GPa), Young’s modulus Y (GPa), bulk modulus B (GPa), shear modulus G (GPa), B/ G ratio, Poisson’s ratio (ν) and cauchy pressure (C_P_) of compound InGeX_3_ (X = Cl, Br).

Compounds	C_11_	C_12_	C_44_	Y	B	G_V_	G_*R*_	B/G	ѵ	Cp
InGeCl_3_	62.70	10.99	10.18	38.11	28.22	13.44	16.45	1.88	0.27	0.80
InGeBr_3_	41.92	14.42	13.85	34.67	23.58	13.81	13.81	1.70	0.26	0.56

Table 2 shows the values for bulk modulus(B), Voigt’s shear modulus(G_V_), Reuss’s shear modulus(G_R_), shear modulus(G), Young’s modulus(Y), and Poisson’s ratio(υ). These values can be determined by applying the following formula^54,55^:

2 $$\:A=2\frac{{C}_{44}}{{C}_{11}-{C}_{12}}$$

3 $$\:\text{B}=({C}_{11}+{C}_{12})/3$$

4 $$\:\text{G}=({\text{G}}_{\text{V}}+{\text{G}}_{\text{R}})/2$$;

5 $$\:{\text{G}}_{\text{V}}=({C}_{11}-{{C}_{12}+3G}_{44})/5$$

6 $$\:{\:\text{G}}_{\text{R}}=\left\{5{C}_{44}\left({C}_{11}-{C}_{12}\right)\right\}/\{4{C}_{44}+3\left({C}_{11}-{C}_{12}\right)\}$$

7 $$\:\text{Y}=9\text{B}\text{G}/(3{\text{B}}_{0}+\text{G})$$

8 $$\:v=(3{\text{B}}_{0}-\text{Y})/6{\text{B}}_{0}$$

9 $$\:{C}_{P}={C}_{12}-{C}_{44}$$

B is the bulk modulus, which is a gauge of a material’s resistance to pressure. The material’s ability to tolerate deformation is assessed by its bulk modulus; a larger bulk modulus denotes a more marked resistance to pressure-induced distortion. Based on Table 2, the bulk modulus of InGeCl_3_ is the greatest, signifying that it has the strongest resistance to volume change under pressure. Similarly, the association between the shear modulus (G) and the material’s reaction to shear stress is portrayed as the ratio of shear stress to shear strain. The response of the material under shear stress within the elastic deformation limit is assessed. Equations (4)–(6) may be employed to identify it, where G_V_ and G_R_ refer to Voigt’s and Reuss’s shear moduli, respectively. Higher values of the shear modulus G reflect greater resistance to deformation. The magnitude of the shear modulus G refers to the material’s aptitude to resist shear strain. The findings suggest that compared to InGeBr_3_, InGeCl_3_ will have a significantly greater resistance to deformation. We can determine the value of Young’s modulus(Y) by applying Eq. (7), which connects Young’s modulus(Y) to the bulk modulus(B) and the shear modulus(G). The material’s stiffness can be inferred from the value of Young’s modulus Y. The higher the Young’s modulus, the less prone to deformation and the more robust the material. The Poisson’s ratio (υ) characterizes a material’s transverse deformation under tensile or compressive stresses. Throughout the elastic constant range of 0.25 to 0.50, the bigger the Poisson’s ratio, the more flexible the material is, presenting that the forces between the material’s atoms are considerable. It is feasible to assess the malleability, ductility, or brittleness by computing Cauchy’s pressure (Eq. 9) and Pugh’s ratio (B/G, limiting value = 1.75)^56^. The conclusions are displayed in Table 2, where it is apparent that both alloys have B/G ratios larger than 1.75 and that their C_P_ values are positive, both of about the alloys’ ductility.

### Electronic properties

**Fig. 3:**
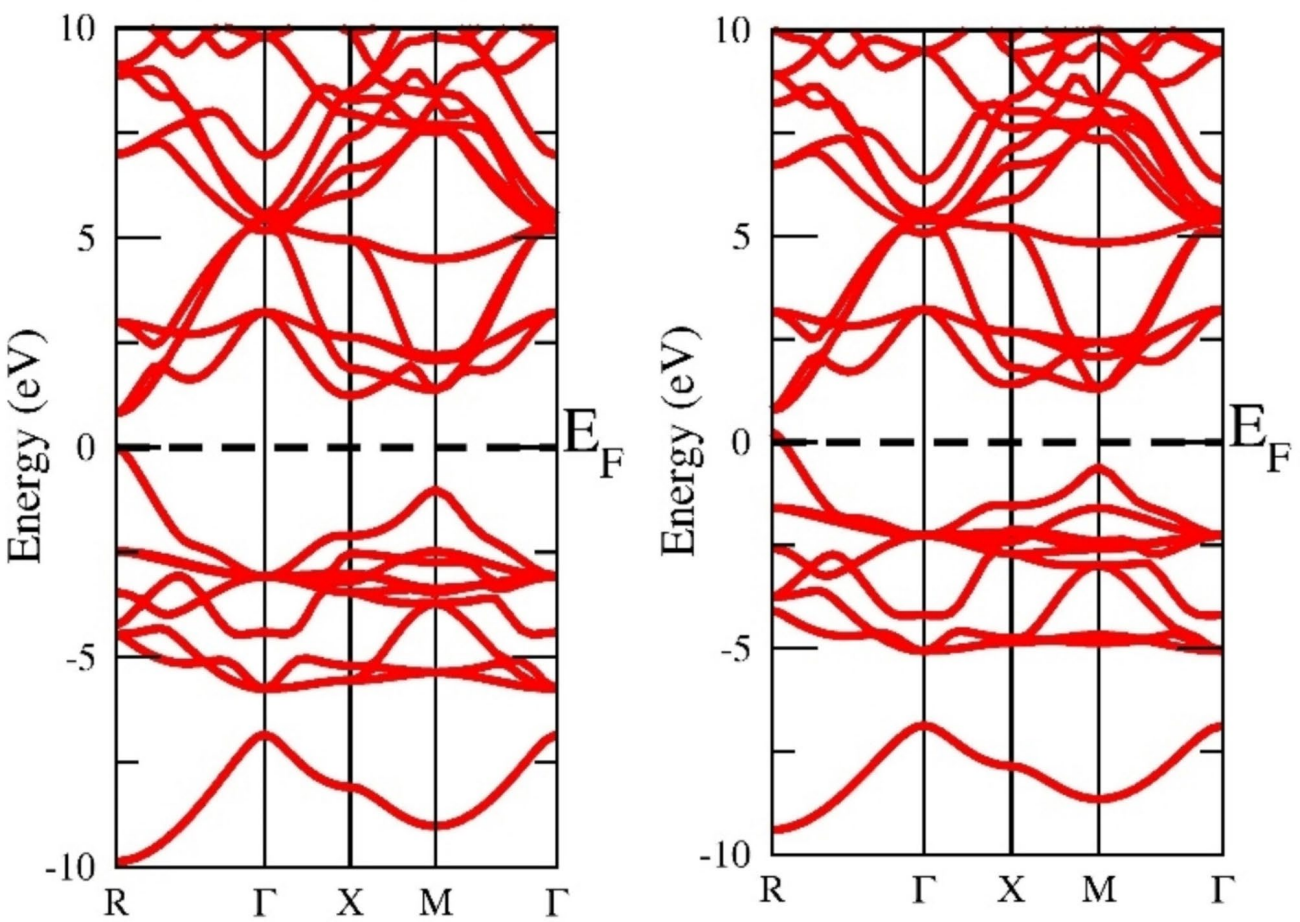
LDA band approximation of InGeX_3_ (X = Cl, Br).

**Fig. 4:**
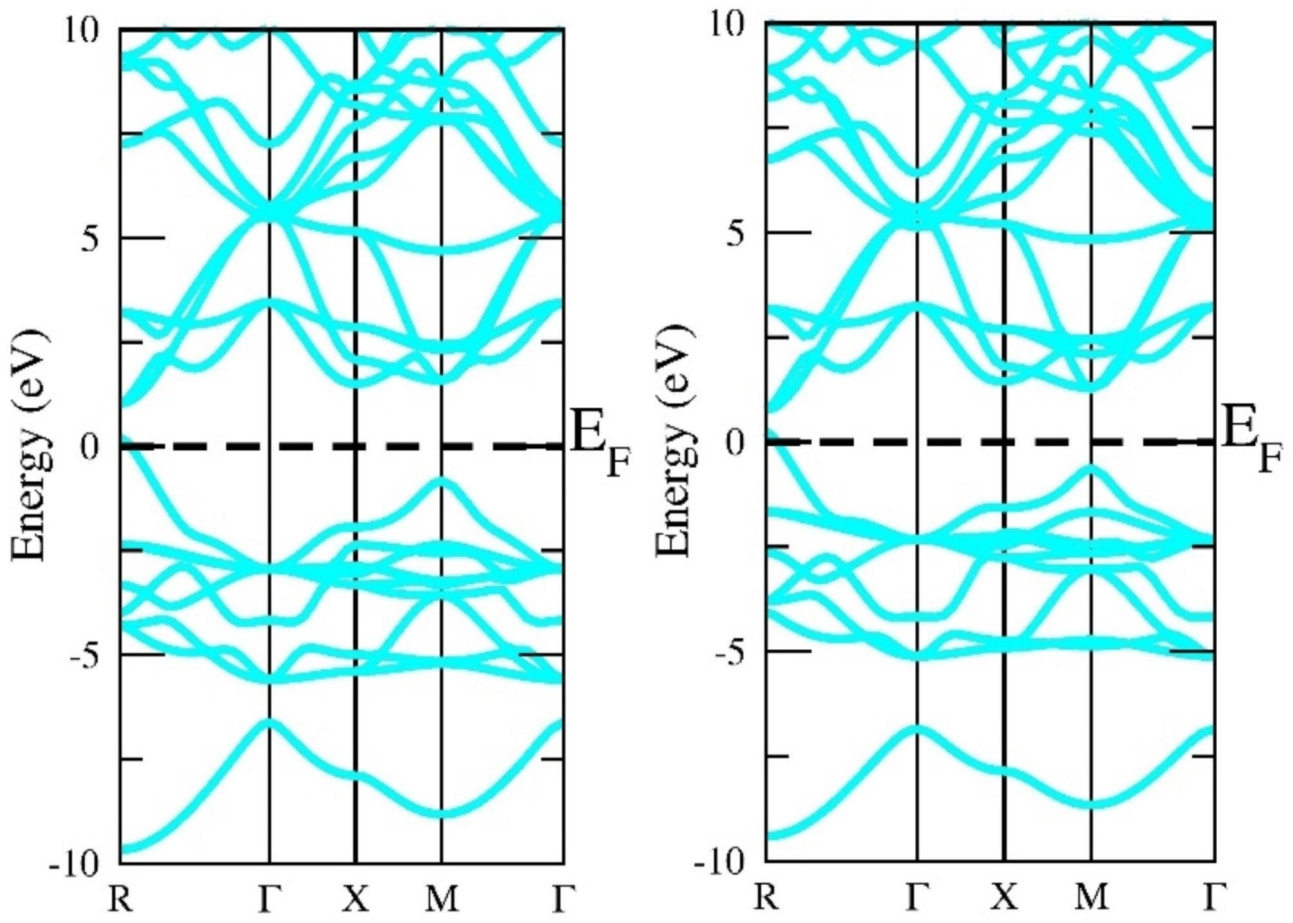
Wu-Cohen-Generalized Gradients Approximation band structure of InGeX_3_ (X = Cl, Br).

**Fig. 5:**
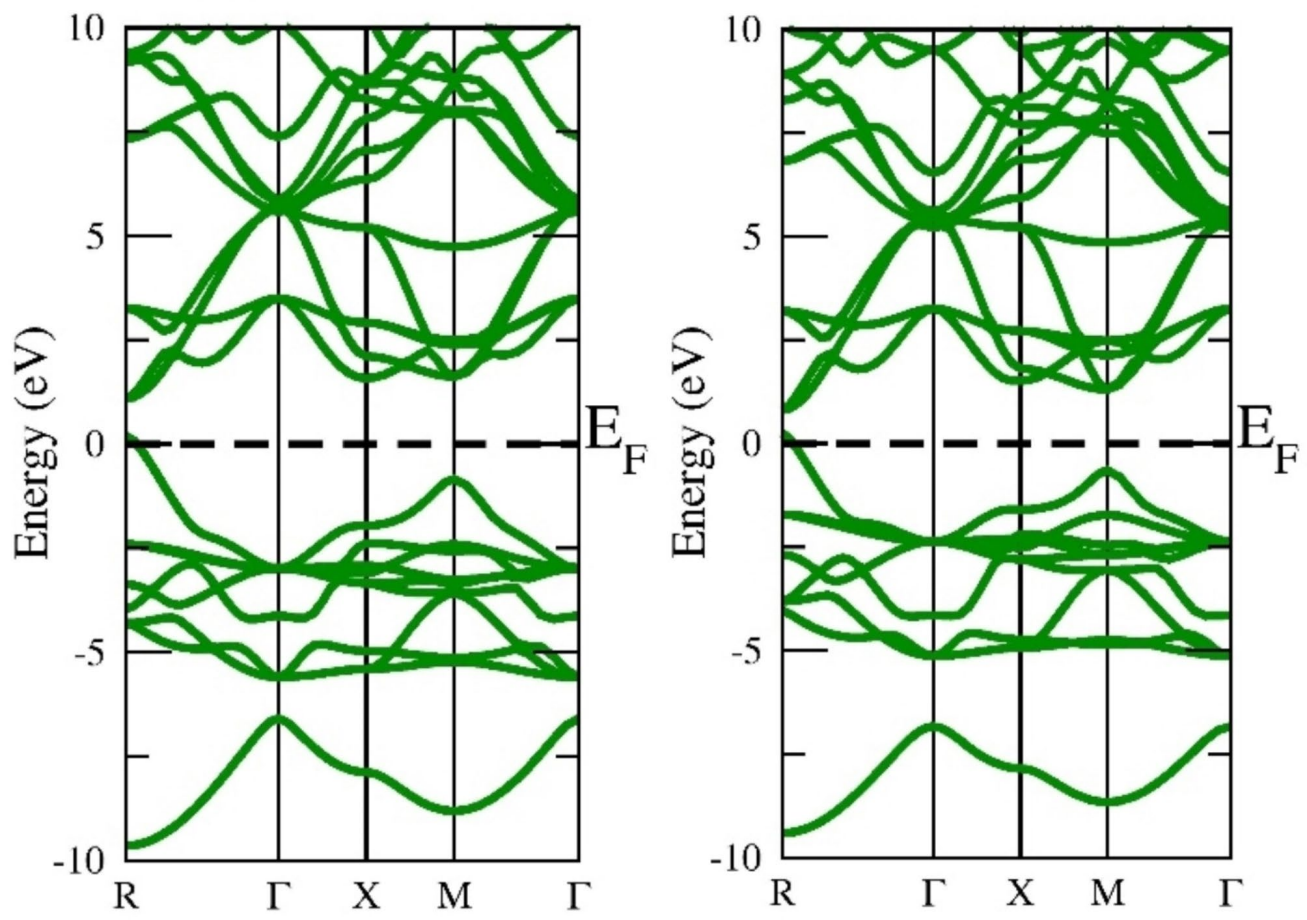
Generalized Gradients Approximation band structure of InGeX3 (X = Cl, Br).

**Fig. 6:**
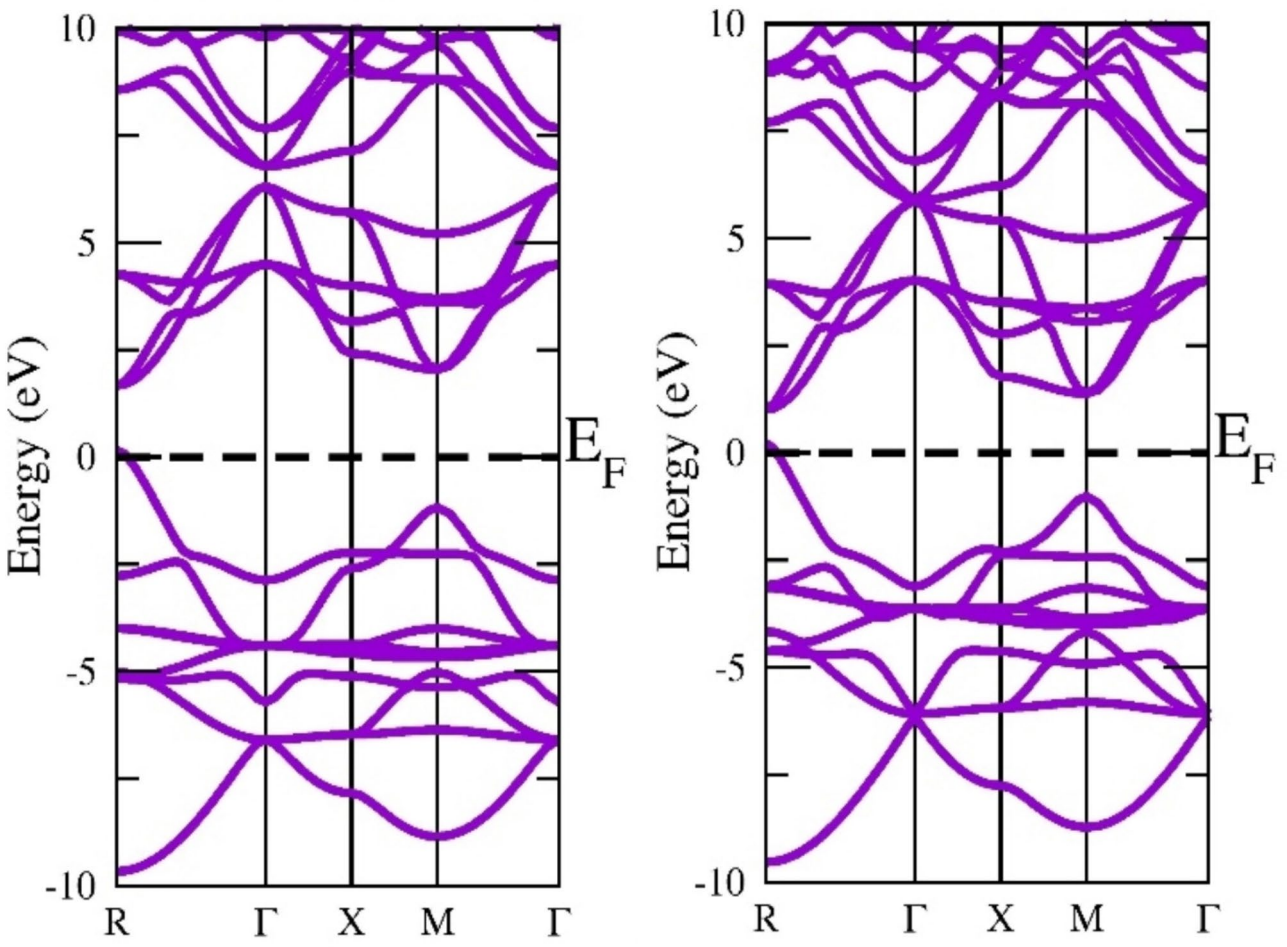
GGA + mBJ band structure of InGeX3 (X = Cl, Br).

The electronic structure is a very effective parameter for describing the nature of the material. If the electronic states in the valence band and conduction band do not overlap at the fermi level the material is a semiconductor else conductor. The electronic states exist a gap at the fermi level separating the valence band and conduction band suggesting the semiconducting nature. In our case, the top of the valence band and the bottom of the conduction band lie on the same plane so there exists a direct band gap at the M symmetry point. If the top of VB and bottom of CB do not lie at the same plane the material is else indirect band gap material. The direct band gap materials are very promising for photovoltaic applications. The more reliable ground state energy and band gap in the current study were achieved by employing the local density approximation (LDA), Generalized Gradient Approximation programmed by Wu-Cohan (WC-GGA), and Perdew-Burke-Ernzerhof (PBE-GGA) exchange-correlation potential functionals. Exchange-correlation potential functionals have been emulated by adopting the semi-local Tran-Blaha modified Becke-Johnson (TB-mBJ) exchange and correlation potential. DFT is based on the basic premise that a system that interacts with fermions can be characterized via its electron density rather than the many-body wave function. The primary hurdle with DFT is that, aside from the free electron gas, the precise function for exchange and correlation is unknown. However, approximations persist, allowing for extremely accurate computation of some physical quantities. Approximations must therefore be adopted. Thus, the authors employed the potential functionals (GGA + mBJ) to execute SCF calculations to identify the electronic band structure and band gap. The electronic band gap of semi-conducting materials is understated by LDA and GGA functionals; as a result, the TB-mBJ technique has been implemented to upgrade its accuracy. The identified band structure curves among wave vector “k” and the energy function for this material in the first Brillouin zone, when deploying this method to three different potentials, which are shown in Figs. 3, 4 and 5, and 6. The estimated band gap values calculated through multiple approximations are displayed in Table 3. By applying different approximations, we have noticed that all of them follow the semiconducting nature of the materials, but the only difference is in their band gap values.

**Table 3:** Calculated band gap of InGeX3 (X = Cl, Br) using LDA, WC-GGA and GGA + mBJ approximations.

Compounds	LDA	WC-GGA	GGA	GGA + mBJ
InGeCl_3_	0.824	1.098	1.157	1.520
InGeBr_3_	0.864	0.853	0.887	0.981

**Fig. 7:**
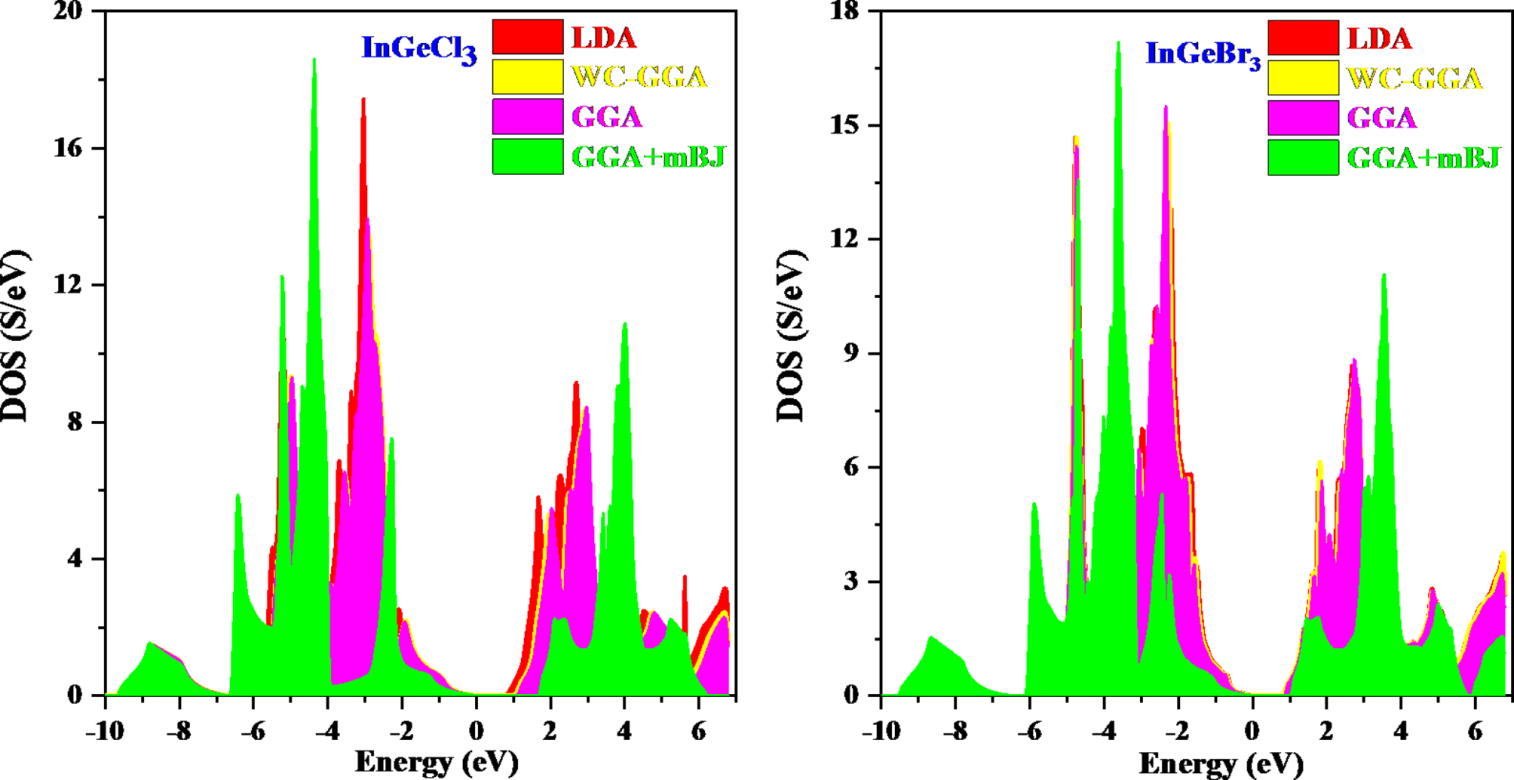
Total density of states of compound InGeX_3_ (X = Cl, Br) using LDA, WC-GGA, GGA and GGA + mBJ approach.

Figure 7 display the plotted Total Density of States (TDOS) and Partial Density of States (PDOS){supplementary} of InGeX_3_ (X = Cl, Br) perovskites via numerous approximations. Of all the compounds under inquiry, the In-6p orbital contributes the most to PDOS in the conduction band. Ge-4p orbitals come next, while halide anion orbitals (Cl-3s and Cl-3p; Br-4s and Br-4p) come last. Conversely, anion p-orbitals (Cl-3p, Br-4p) predominate in regions close to the top of the valence bands, followed by Ge- and In-orbitals. The fermi level separates the valence band and conduction band which is set at 0 eV energy. The electronic states in the valence band and conduction band do not cross the fermi level or the electronic states in the valence band and conduction band form a gap at the fermi level, thus resulting in the semiconducting nature of the materials.

### Optical properties

The optical properties of InGeX_3_ (X = Cl, Br) were looked into for incident photon energy spanning 0 to 13 eV. Optical characteristics pertain to a material’s behavior towards incident light. Optical properties comprise dielectric constant, refractive index, optical conductivity, reflectivity, refractive index, energy loss, and absorption coefficient. The complex dielectric function assesses the optical properties of multiple materials^57–63^.

**Fig. 8:**
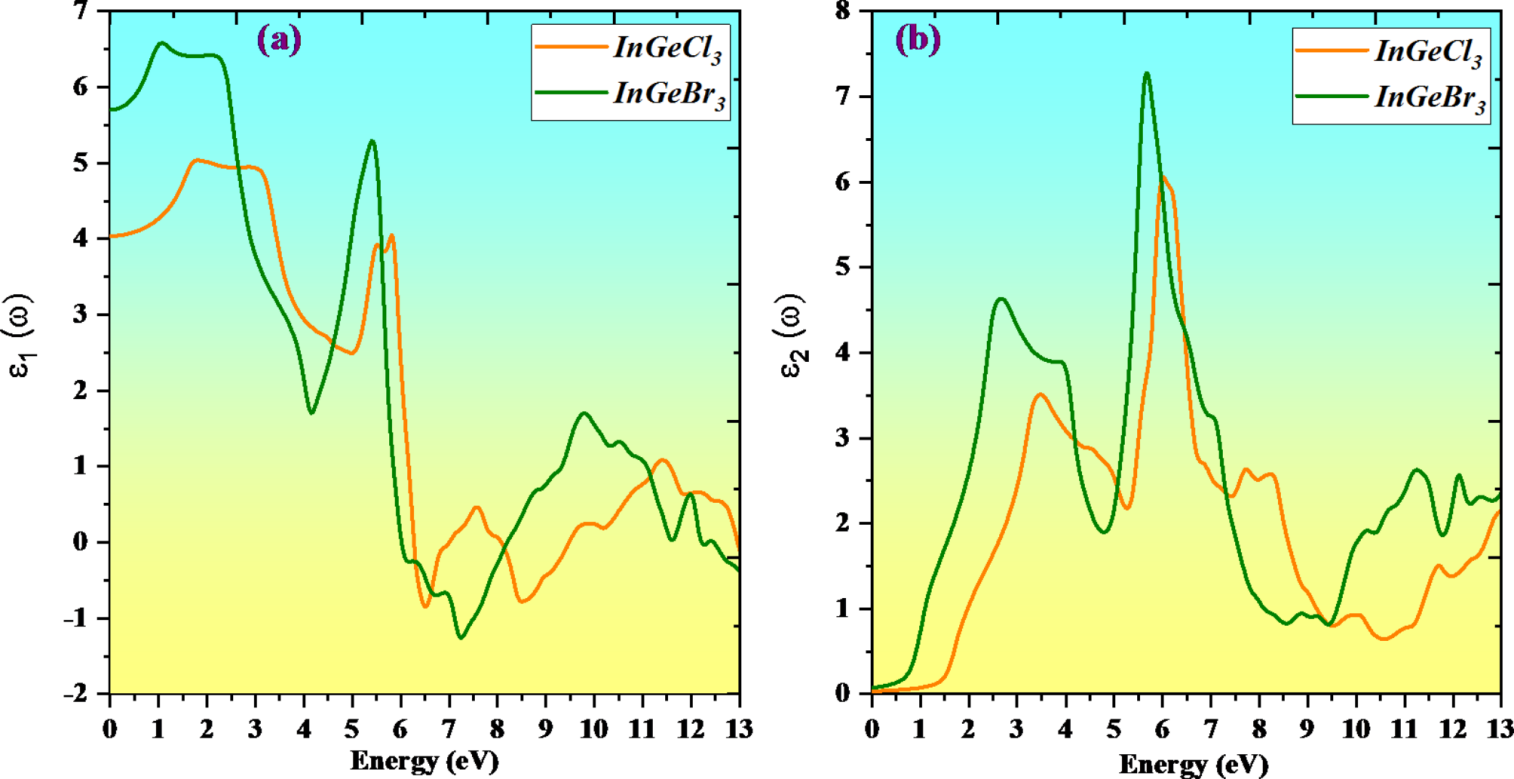
Variation of optical properties with a real (ε1(ω)) and imaginary (ε2(ω)) dielectric coefficient of double perovskites InGeX_3_ (X = Cl, Br) compounds.

The method by which a material adapts to electromagnetic radiation is explained by its dielectric function. Since the optical properties are frequency-dependent and in a specific way interconnected, the dielectric function is a parameter that dictates how all other characteristics can be explored. ε (ω) = ε_1_(ω) + i ε_2_(ω) is the complex dielectric function with real ε_1_(ω) and imaginary ε_2_(ω) components. The dispersion tendency and the polarization character of the material are addressed by the real part ε_1_(ω). The material’s potential for absorption is linked to the imaginary component ε_2_(ω). The dielectric constant in the energy range of 0 to 13 eV is displayed in Fig. 8. As illustrated in Fig. 8a, the static dielectric constants of InGeCl_3_ and InGeBr_3_ are projected to be 4.01 and 5.74, respectively, when they dwell at zero energy. The static value of the dielectric function clarifies the degree of charge recombination, which is equivalent to the functionality of optoelectronic devices. A material with a greater dielectric function will have a lesser charge recombination rate. It will enhance optoelectronic device performance. As the value passes into the UV spectrum, it then commences to gently drop. The occurrence leads to negative values of the dielectric function’s real component in this region. These substances have negative values between 6 and 13 eV. Due to their numerous spikes in the visible and UV domains, all of the materials repeatedly acquire negative values. The alteration in ε_2_(ω) with the energy of the incoming radiation is evident in Fig. 8b. Its peaks assist in detecting the energy loss in these compounds by clarifying the transition between the valence band maxima and conduction band minima. It symbolizes the radiation ingested by the compound. The specific absorption peaks can be seen in the InGeCl_3_ and InGeBr_3_ imaginary parts at energies of 5.75, and 5.99 eV.

**Fig. 9:**
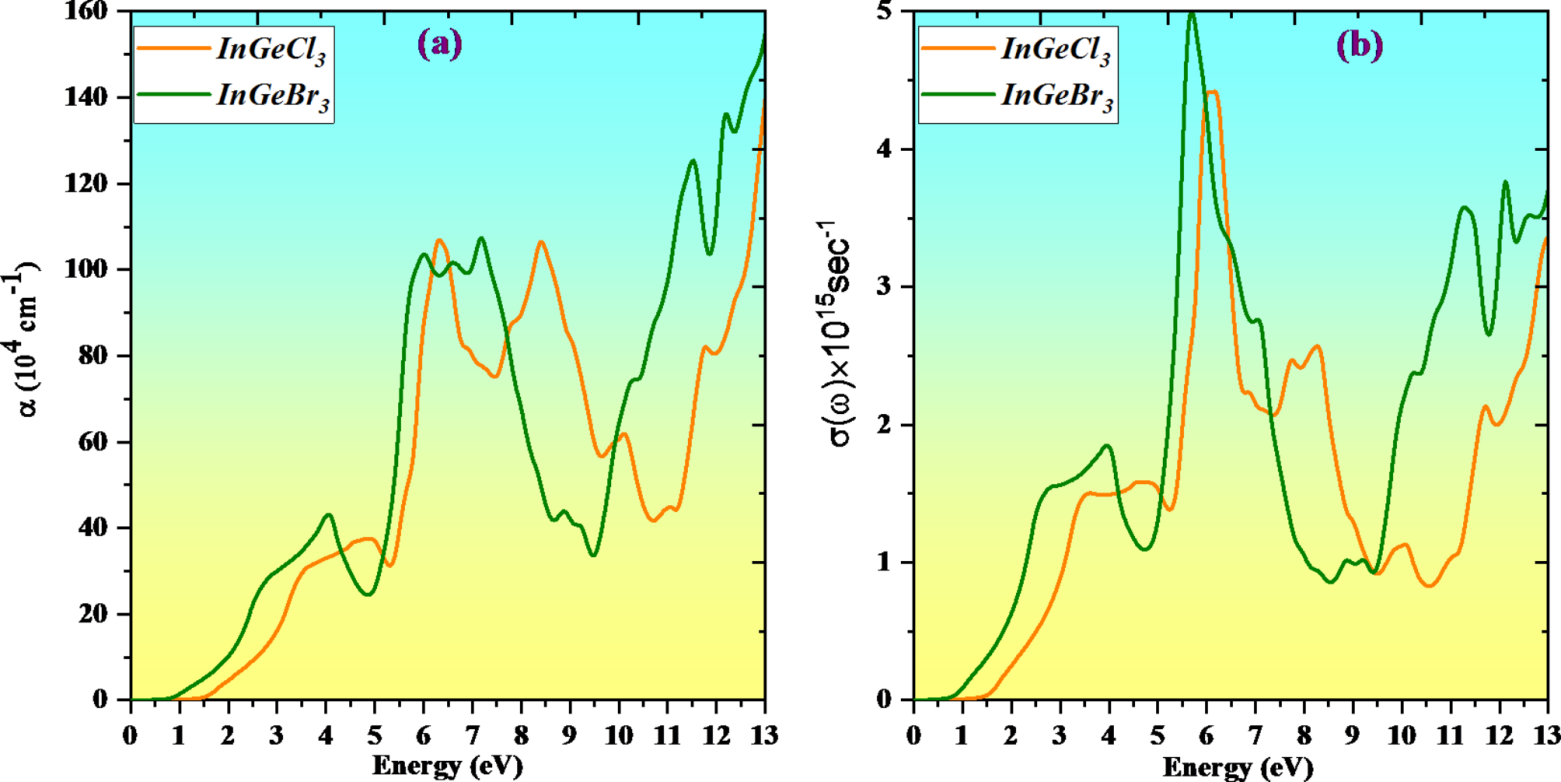
Variation of absorption coefficient (α(ω)) and optical conductivity (σ(ω)) with a photon energy of InGeX_3_ (X = Cl, Br) compounds.

The absorption coefficient is an essential and vital parameter that delivers valuable information about the amount of optical energy absorbed per unit length^33–64^. Optical absorption happens when an atom’s transition frequency and the frequency of an incoming photon interact. Materials can capture photons of a particular frequency since the absorption coefficient fluctuates with frequency. Electrons migrate from occupied higher valence band states to easily accessed unoccupied states in the lower conduction band, leading to optical absorption. Every semiconductor material possesses a threshold for absorption of light below which it ceases to function. Above this threshold, the photon can interfere with valence electrons and may ingest light. The relationship α = 4πk/λ symbolizes the maximum rate of light decay. The determined values of the absorption coefficient α(ω) for multiple compounds are displayed in Fig. 9a. The absorption rate for InGeX_3_(X = Cl, Br) commences at about 1 eV. For InGeCl_3_ and InGeBr_3_, the greatest elevation values are approximately 12.5 eV. For any given frequency, the association between the induced current density and the created electrical field strength in a material is outlined by its optical conductivity (see Fig. 9b). The definition of optical conductivity, which is the broadening of electrical transport to optically high-energy incident photons, is the conduction of electrons propelled by an imposed electromagnetic field, as determined by the complex dielectric function. The perovskite materials InGeCl_3_ and InGeBr_3_ exhibit high optical conductivity at 6.10 and 5.76 eV, respectively, when exposed to an incident energy.

**Fig. 10:**
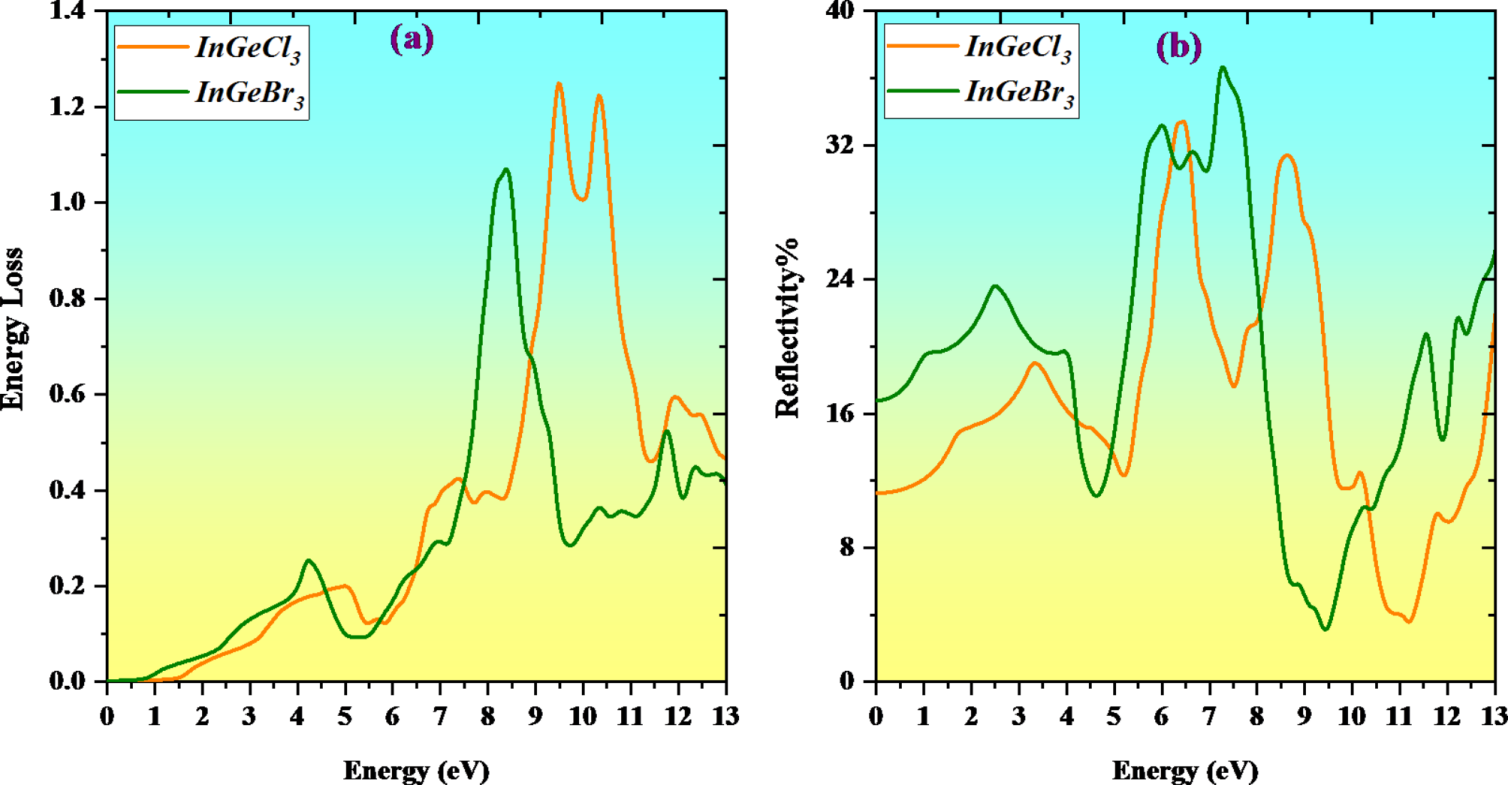
Variation of Energy Loss and Reflectivity with a photon energy of InGeX_3_ (X = Cl, Br) compounds.

Since photons dissipate energy when interacting with matter and light, the energy-loss function L(ω) is available to assess this energy loss. Figure 10a shows the numerical values of L(ω) and the associated pattern as a function of energy (eV). It turns out that, despite the negligible increase in photon energy, the energy-loss function’s value increases. The Energy loss curve glides down the X-axis to the band gaps for Cl (1.52 eV) and Br (0.98 eV). After that, it begins ascent and approaches its peak values of 1.25 and 1.09 for Cl and Br at 9.5 and 8.49 eV, respectively. Figure 10b displays the compounds’ reflectivity R(ω), demonstrating that all materials have a fairly modest reflectivity over the entire energy range taken into account. With precise values anticipated to be 33.75% at 6.49 eV for InGeCl_3_ and 36.78% at 7.48 eV for InGeBr_3_, the compounds’ maximum reflectivity levels are about 36%. These comparatively low reflectance values highlight the materials’ performance as absorption by suggesting negligible light loss via light absorption^65^.

**Fig. 11:**
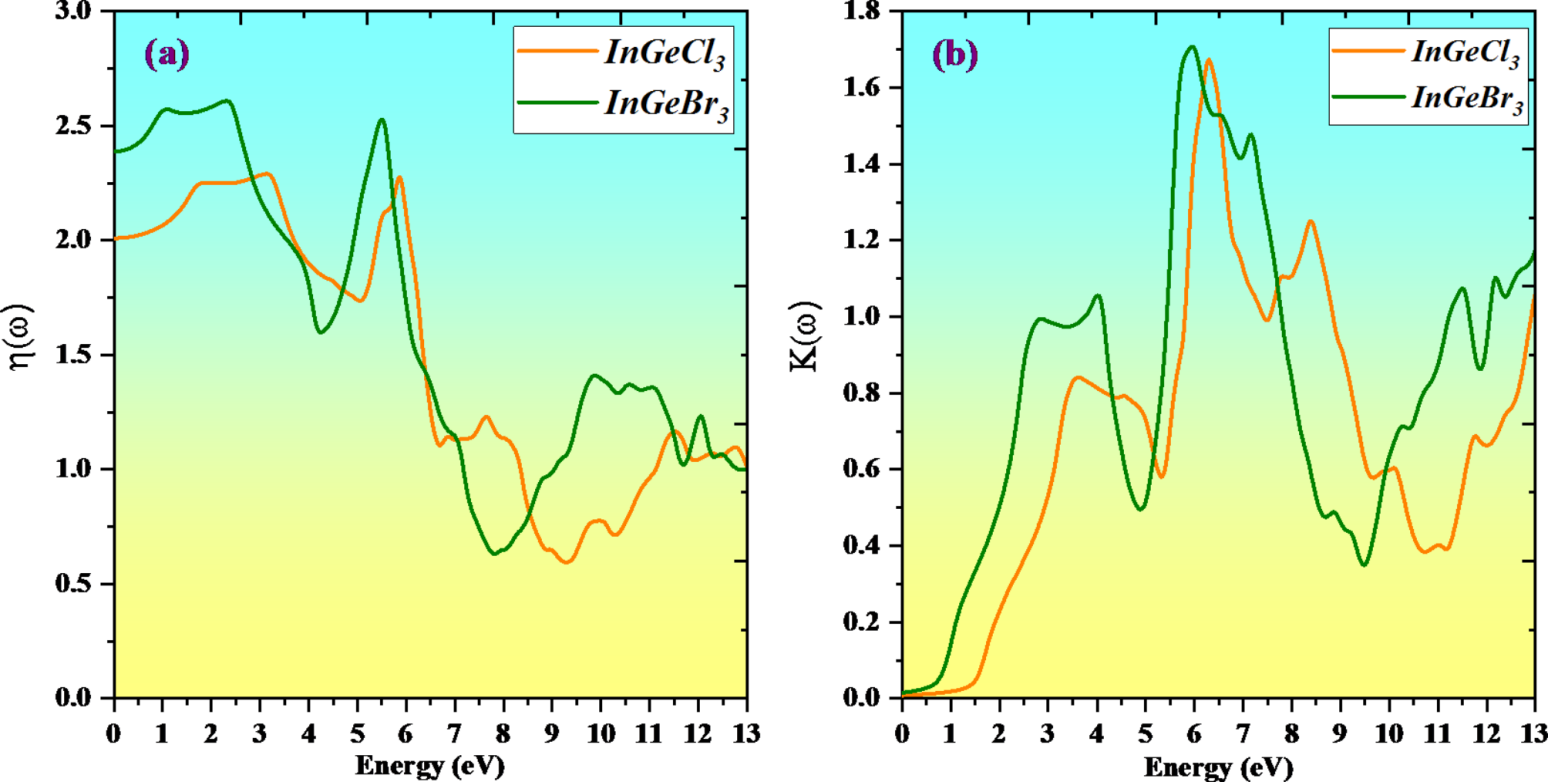
Variation of Refractive index n(ω) and Extinction coefficient K(ω) with a photon energy of compounds InGeX_3_(X = Cl, Br) respectively.

The refractive index of a material is an index of how light traverses it. Light travels less rapidly when materials have greater refractive indices, altering the direction of the light inside the material correspondingly more. Since the refractive index (n(ω)), is so beneficial to photoelectric gadgets, comprehending it is indispensable when assessing the degree of refraction. The determined refractive index for the key compounds is shown in Fig. 11a. InGeCl_3_ has a maximum refractive index of 2.29 at 3.10 eV, whereas InGeBr_3_ has a maximum refractive index of 2.62 at 2.48 eV. The quantity of light refracted as it travels through a material is identified by its refractive index^66^. A greater quantity of light is refracted by larger factor values. In simple terms, any approach that raises a material’s electron density also raises its refractive index. The extinction coefficient is a parameter that specifies the degree to which a material absorbs or reflects radiation or light at a particular wavelength. The outcome extinction coefficient k(x) for InGeX_3_ (X = Cl and Br) compounds is shown in the Fig. 11b. At 6.49 eV, InGeCl3’s localized maximum extinction coefficient is approximately 1.68, while InGeBr_3_ is approximately 1.71 at 6.0 eV.

### Thermodynamic properties

**Fig. 12:**
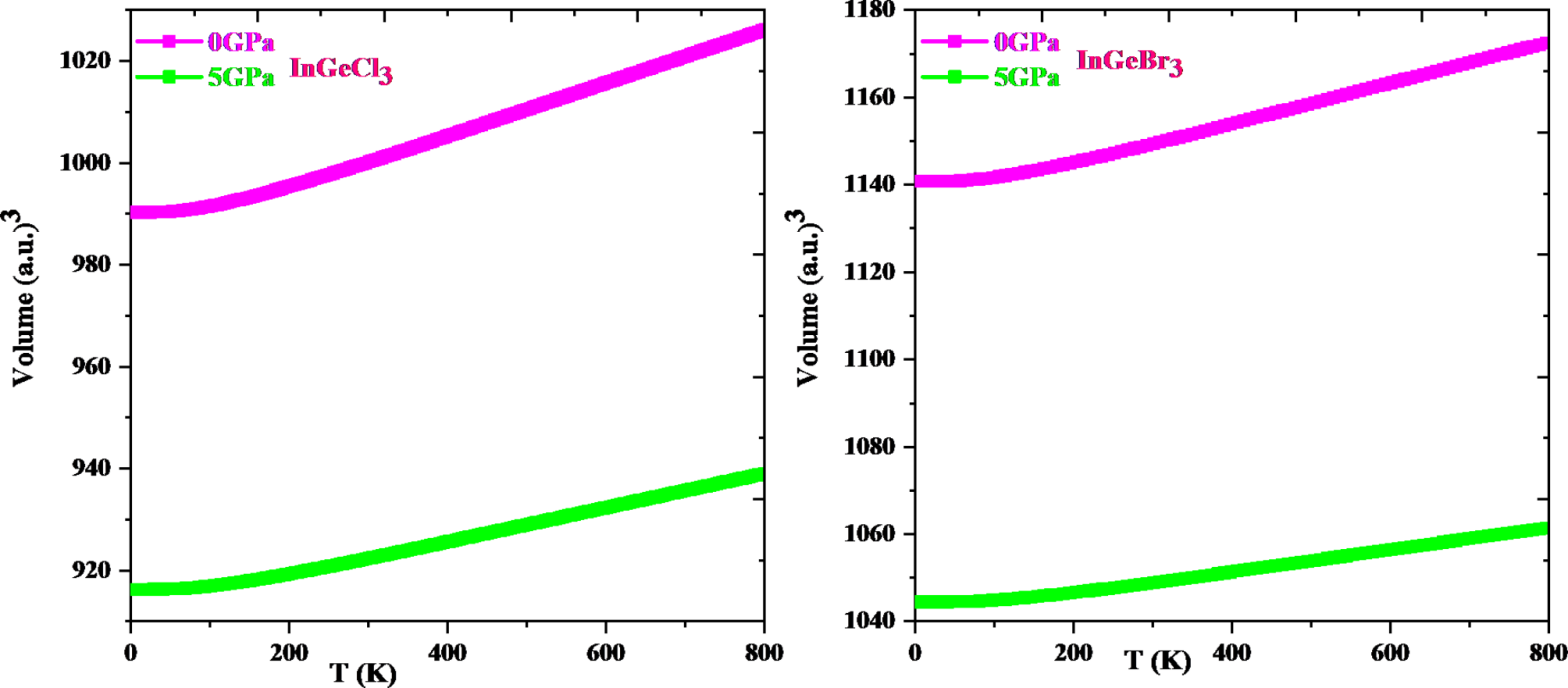
Volume as a function of temperature and pressure for InGeX_3_ (X = Cl, Br) respectively.

**Fig. 13:**
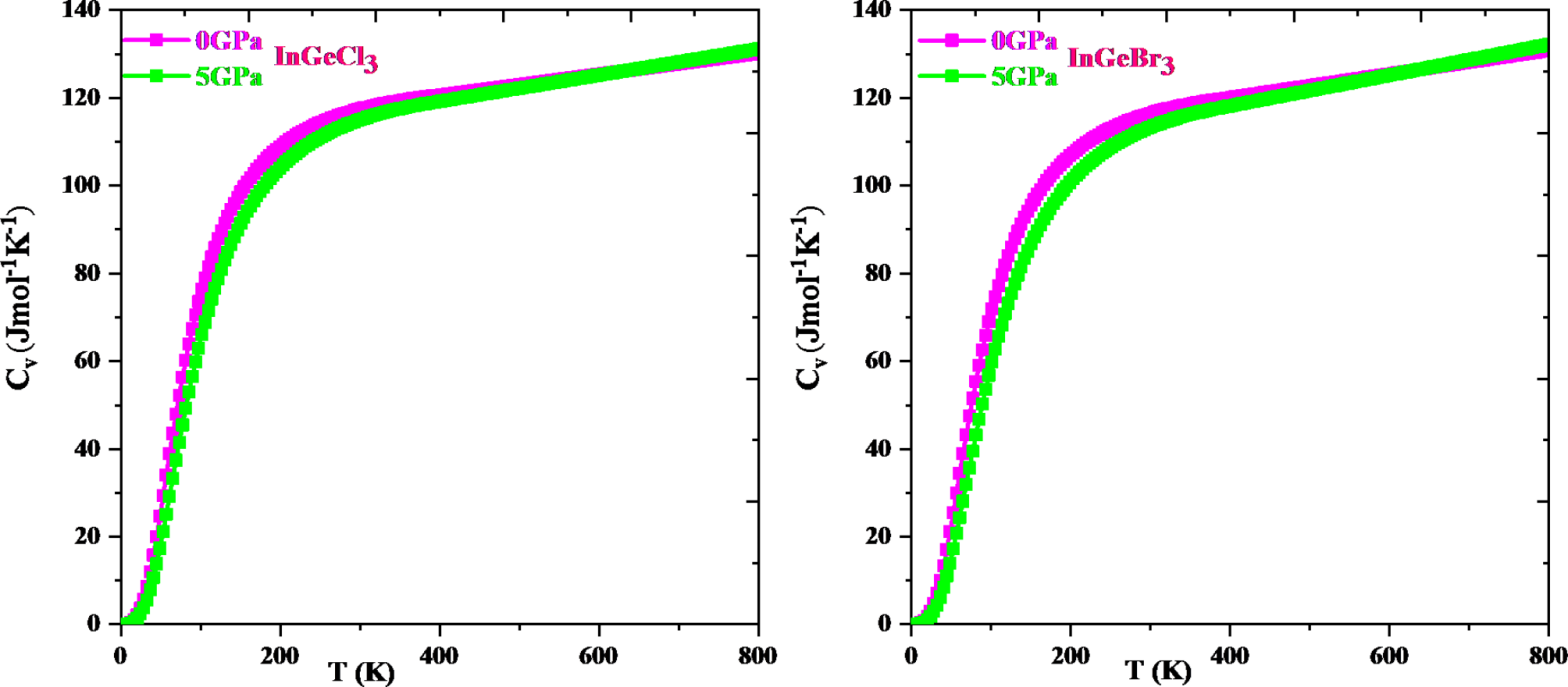
Specific heat capacity (Cv) as a function of temperature and pressure for InGeX_3_ (X = Cl, Br) respectively.

**Fig. 14:**
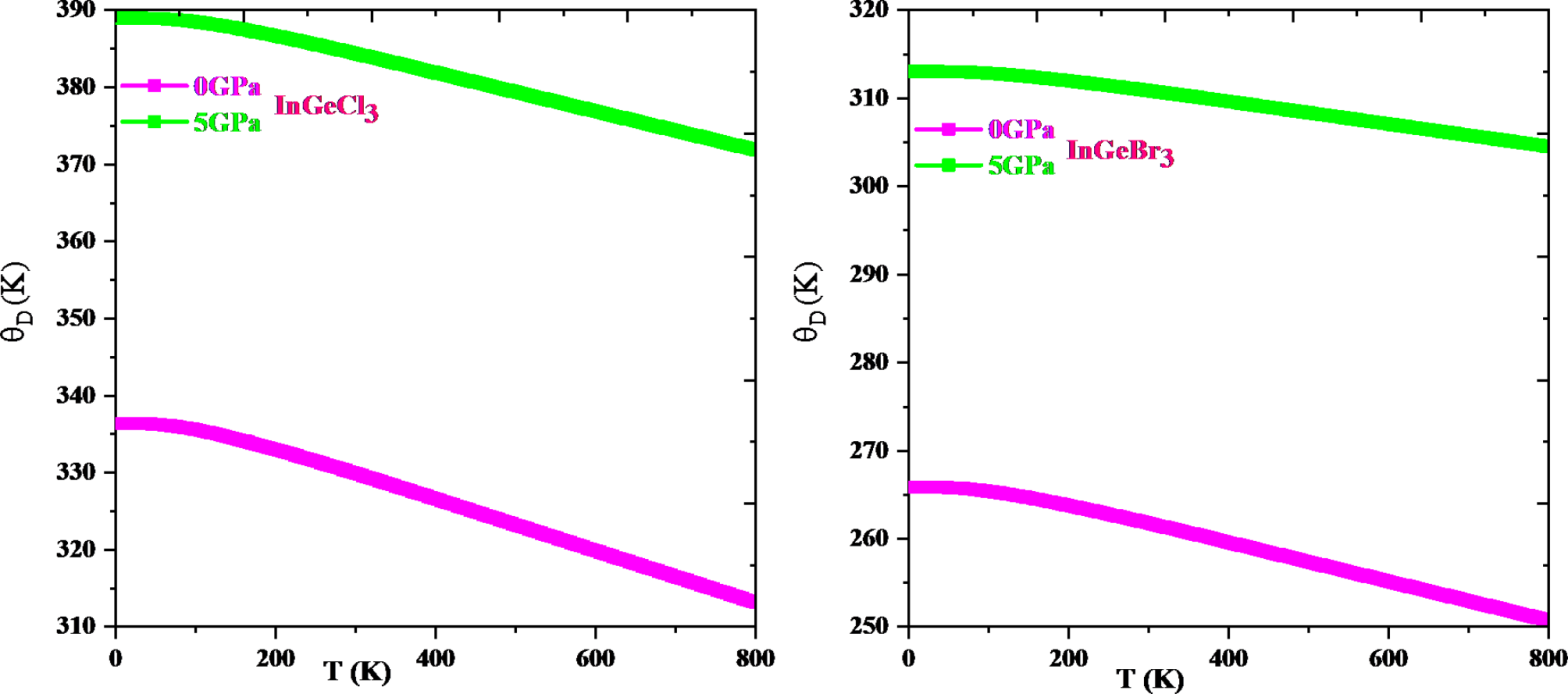
Debye Temperature (θ_D_) as a function of temperature and pressure for InGeX_3_(X = Cl, Br) respectively.

**Fig. 15:**
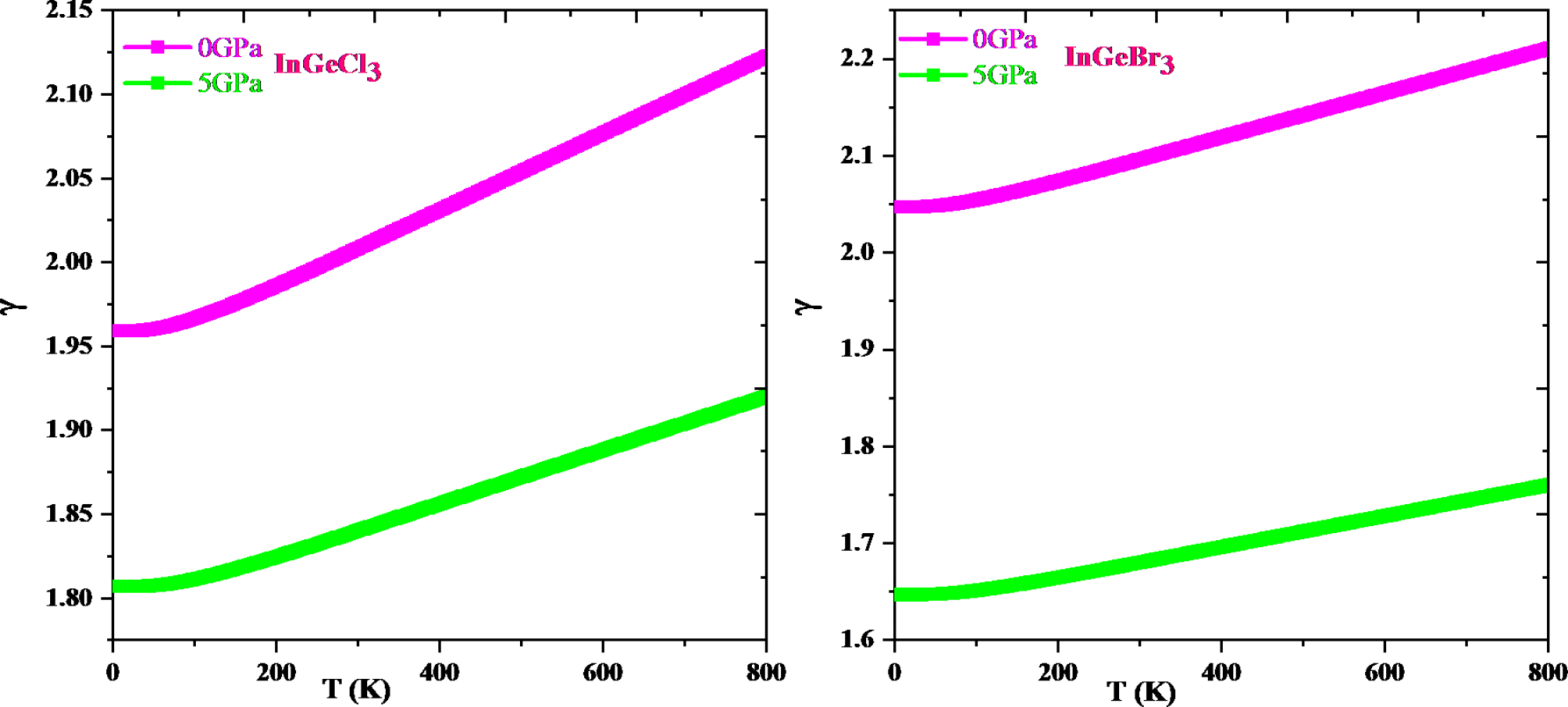
Gruneisen parameter (γ) as a function of temperature and pressure for InGeX_3_ (X = Cl, Br) respectively.

Thermodynamic properties offer extensive knowledge about a material’s activity under diverse pressure and temperature conditions and permit us to assess a material’s exact performance. In-depth information about significant material properties like interatomic interaction and thermodynamic stability can be obtained via thermodynamic parameters like heat capacity, Debye temperature, and the grüneisen parameter. The resulting knowledge can then be utilized to direct the application of materials and the construction of gadgets. This research explores how temperature and pressure affect thermodynamic parameters such as volume (V), specific heat (Cv), Debye temperature (θD), and Grüneisen parameter (γ) adopting the quasi-harmonic Debye approximation. The temperature ranges from 0 to 800 K, while the pressure ranges from 0 to 5 GPa. Figure 12 shows that, for a specific pressure value, cell volume expands with rising temperature; conversely, it diminishes with growing pressure. This type of action is quite prevalent in solids, and it makes logic that a solid expands with temperature and contracts under pressure. It is evident from Fig. 13 that the heat capacity for both compounds increases quickly up to 400 K at constant volume and then gradually at higher temperatures. It adheres to the well-known Dulong-Petit law^67^, which is acceptable to all solids over 900 K. InGeCl_3_ and InGeBr_3_ have Dulong-Petit limits of around 133.51 J mol^− 1^ K^− 1^ and 132.40 J mol^− 1^ K^− 1^, respectively. The Debye temperature variation is seen in Fig. 14 and the Debye temperature tends to rise with rising pressure. Conversely, the decline in Debye temperature is established with a temperature rise, but at a slower rate than the increase with increasing pressure. An alteration in a lattice’s vibrational frequency triggered by variations in pressure and temperature is summed up by the Grüneisen parameter (γ)^68^. Figure 15 depicts the fluctuation of (γ) for, InGeCl_3_ and InGeBr_3_ respectively. In InGeCl_3_ and InGeBr_3_, the variation exhibits a similar trend. The (γ) value elevates as temperature rises, but pressure has an adverse effect; as pressure rises, the (γ) value decreases.

### Thermoelectric properties

Thermoelectric materials, which induce electrical energy across temperature variations, are vital for reusing and producing energy deploying renewable resources. As a result, it is of the utmost significance to comprehend the transport characteristics of halide single perovskites InGeX_3_ (X = Cl, Br) while fabricating devices. One widely recognized computational method for assessing a material’s transport behavior over a wide range of frequencies is the BoltzTraP code. In the 0–1000 K temperature range, the thermoelectric efficiency of InGeX_3_ (X = Cl, Br) is fully assessed employing the following metrics: power factor, Seebeck coefficient (S), electrical conductivity (σ/τ), and thermal conductivity (ke/τ)^54,69,70^.

**Fig. 16:**
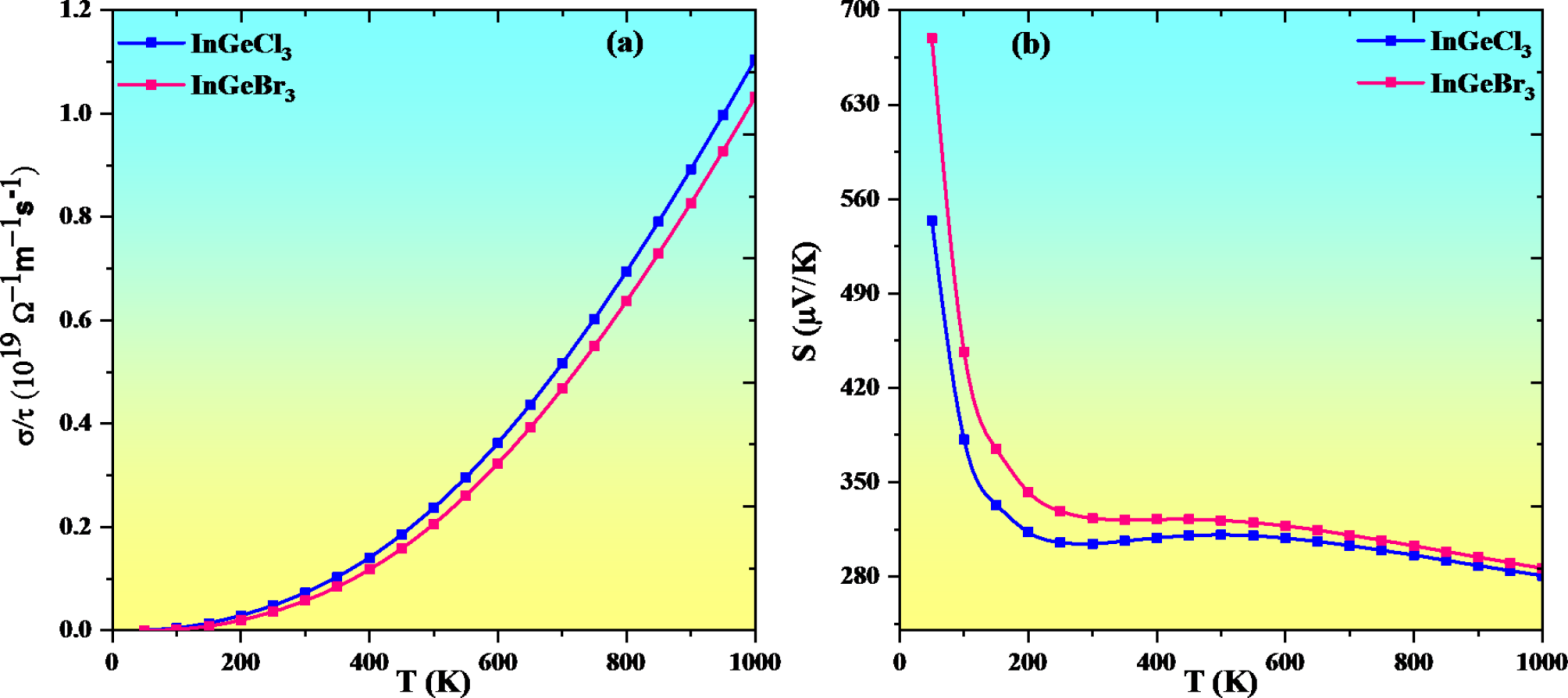
Variation of Seebeck coefficient and electrical conductivity with temperature For InGeX_3_ (X = Cl, Br).

The growing temperature of semiconductors raises conductivity due to an adequate supply of charge carriers, which entails transporting electrons from VB to CB. Electrical conductivity advances gradually until it reaches 1000 K (see Fig. 16a). The rising conductivity validates these compounds’ semiconducting properties. Equation *σ = neμ* states that charge carrier concentration improves with temperature, increasing electrical conductivity. The variables σ, n, e, and µ stand for electrical conductivity, charge carrier concentration, an electron’s charge, and mobility, respectively. The rise in electrical conductivity with temperature clarifies the negative temperature coefficient of resistance that reflects the semiconducting characteristic of these materials InGeX_3_ (X = Cl, Br). At 300 K(1000 K) the value of electrical conductivity for InGeCl_3_ and InGeBr_3_ are 0.072(1.10 × 10^19^ W^−1^m^−1^s^− 1^) and 0.057 (1.03 × 10^19^ W^−1^m^−1^s^− 1^) respectively.

The Seebeck coefficient (S) or thermoelectric power is the ratio of the difference in voltage across the junction (∆*V*) to the difference in temperature (∆*T*), which is described by the intrinsic properties of the material, and it can be mathematically expressed as S = $\:\frac{-{\Delta\:}\text{V}}{{\Delta\:}\text{T}}$ ; where the negative sign indicates that the voltage developed at the junction always opposes the temperature difference, this is the principle behind describing the thermoelectric power generator. From Fig. 16b it is clear that the Seebeck coefficient decreases with an increase in temperature which is the property of a semiconducting material. The material’s ability to generate induced EMF when a temperature differential is enforced is identified as the Seebeck coefficient. The Seebeck coefficient (S) estimation is shown with T in Fig. 16b. The value of S falls with increasing temperature. S is positive for two materials, so holes comprise the bulk of the charge carriers in those materials. The anticipated values of S at 50 K(1000 K) for InGeCl_3_ and InGeBr_3_ are 544(280 µVK^−1^) and 679(286 µVK^−1^).

**Fig. 17:**
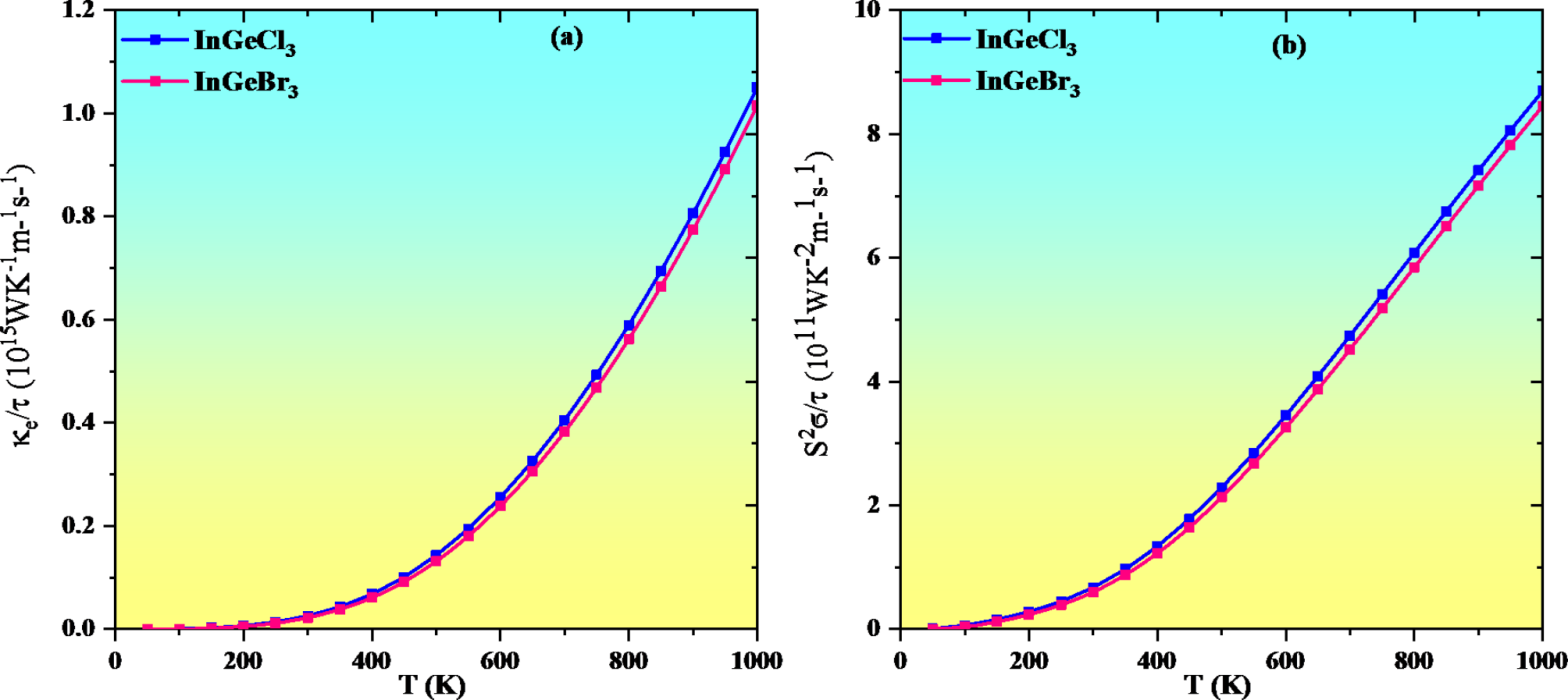
Variation of electronic thermal conductivity and power factor(S^2^σ/τ) with temperature For InGeX_3_ (X = Cl, Br).

Figure 17(a) depicts the determined electronic heat conductivity κe. The degree to which charge carriers assist in heat transport is estimated by this metric. Noted alternatively, it quantifies the degree to which a substance conducts heat. To accomplish excellent thermoelectric efficiency, low κe values must be maintained^71,72^. Figure 17a shows that the κe values of these compounds increase with temperature. Moreover, we have also calculated the power factor as revealed in Fig. 17b. The thermoelectric efficiency can be gauged by computing the power factor (PF = S^2^ σ/τ) that composes the figure of merit expression numerator. Figure 17b depicts that PF increases, as the temperature rises from 0 K to 1000 K, respectively. The increasing PF with increasing temperature indicates that investigated perovskites remain attractive at elevated temperatures due to their potential for wasted heat to electricity conversion applications. The evaluated values are listed in Table 4.

**Table 4:** Computed values of power factor(S^2^σ/τ) and electronic thermal conductivity (κ_e_/τ) with temperature for InGeX_3_ (X = Cl, Br).

Compounds	Electronic thermal conductivity (WK^− 1^m^− 1^s^− 1^)		Power factor (WK^− 2^m^− 1^s^− 1^)	
	300 K	1000 K	300 K	1000 K
InGeCl_3_	0.025 × 10^15^	1.05 × 10^15^	0.067 × 10^11^	8.69 × 10^11^
InGeBr_3_	0.022 × 10^15^	1.01 × 10^15^	0.60 × 10^11^	8.45 × 10^11^

## Conclusion

In the current research, semi-classical Boltzmann and density functional theory (DFT) were implemented to assess the structural, electronic, thermoelectric, and thermodynamic properties of the cubic InGeX_3_ (X = Cl, Br) material. The incorporation of Trans-Blaha modified Becke-Johnson (TB mBJ) approximations in GGA is pointed out to avoid devaluation of the band, whose value of 1.52 eV and 0.98 eV is direct relative to (M–M) symmetric sites for InGeCl_3_ and InGeBr_3_ respectively. Furthermore, centered on the Born stability criteria and Poisson ratio, they are all projected to be mechanically stable and ductile, respectively. Additionally, using the Gibb2 package, thermodynamic characteristics were ascertained and their temperature dependency was discovered. The perovskites’ low reflectance, high absorption, and conductivity all emphasize futuristic advantages in optoelectronics, particularly photovoltaics. The growing PF anticipated opportunities for converting waste heat to energy.

## Electronic supplementary material

Below is the link to the electronic supplementary material.

Supplementary Material 1

## Data Availability

The datasets generated and/or scrutinized during the current study would be available from the corresponding author upon reasonable request.
