# Supplementary material for: Analyzing the structural, optoelectronic, and thermoelectric properties of InGeX3 (X = Br) perovskites via DFT computations
Source: Sci Rep. 2024 Oct 9;14:23575. doi: 10.1038/s41598-024-72745-w (PMC11464498; doi:10.1038/s41598-024-72745-w)
Supplement: Supplementary file 1 — Supplementary Material 1 [file 41598_2024_72745_MOESM1_ESM.docx]

**Supplementary 1:**

**partial density of states of compound InGeX_3_ (X=Cl, Br) using the LDA approach**

**partial density of states of compound InGeX_3_ (X=Cl, Br) using WC-GGA**

**partial density of states of compound InGeX_3_ (X=Cl, Br) using GGA approach**

**partial density of states of compound InGeX_3_ (X=Cl, Br) using the GGA+mBJ approach**
